# Supplementary material for: Functional Role of NOXA in Hypoxia-Mediated PD-L1 Inhibitor Response in Hepatocellular Carcinoma
Source: Int J Mol Sci. 2025 May 16;26(10):4766. doi: 10.3390/ijms26104766 (PMC12112667; doi:10.3390/ijms26104766)
Supplement: Supplementary file 1 [file ijms-26-04766-s001.zip › ijms-3566789-supplementary.pdf]

Supplementary Materials

Table S1. Univariate Cox regression analysis from HHOs

Gene	P-value	HR (CI)
N4BP2L1	5.68×10^{-4}	0.626 (0.48-0.817)
PHLDA2	2.86×10^{-4}	1.24 (1.1-1.39)
CABYR	1.91×10^{-4}	1.3 (1.13-1.49)
AFM	1.88×10^{-4}	0.854 (0.786-0.928)
UPB1	1.82×10^{-4}	0.829 (0.751-0.914)
LMNB1	1.31×10^{-4}	1.37 (1.17-1.61)
CENPM	1.11×10^{-4}	1.34 (1.16-1.56)
POLA1	5.60×10^{-5}	1.89 (1.39-2.57)
KIF11	4.32×10^{-5}	1.53 (1.25-1.87)
BUB1B	1.60×10^{-5}	1.57 (1.28-1.93)
KIF4A	8.27×10^{-6}	1.45 (1.23-1.71)
GINS1	2.24×10^{-6}	1.58 (1.31-1.9)
CCNB1	1.76×10^{-6}	1.46 (1.25-1.7)
HMMR	1.72×10^{-6}	1.6 (1.32-1.93)
SPC25	1.26×10^{-6}	1.71 (1.38-2.13)
NDC80	5.70×10^{-7}	1.67 (1.36-2.04)
TPX2	5.62×10^{-7}	1.46 (1.26-1.69)
DLGAP5	2.00×10^{-7}	1.68 (1.38-2.04)
KIF20A	5.76×10^{-8}	1.65 (1.38-1.98)
CENPA	3.28×10^{-8}	1.72 (1.42-2.08)
TRIP13	1.93×10^{-8}	1.71 (1.42-2.06)

Table S2. LASSO algorithm analysis results

Gene	coefficient
CENPA	0.154873411588668
KIF20A	0.0587895906338177
DLGAP5	0.00907923231906613
HMMR	0.231485339579942
UPB1	-0.0274610875959709
AFM	-0.00394870637764046
CABYR	0.122299886713929
PHLDA2	0.102871162197263
N4BP2L1	-0.185544171498896

Table S3. Cox Regression Analysis for the Hypoxia Risk Scoring Model related to Drug Response Prediction

Gene	log HR	log HR SE	HR	t	p	95% CI Lower	95%CI Upper
PHLDA2	0.1497	0.0753	1.1615	1.9887	0.0467	1.0022	1.3462
DLGAP5	0.0757	0.2695	1.0787	0.281	0.7787	0.636	1.8293
N4BP2L1	-0.2318	0.1636	0.7931	-1.4163	0.1567	0.5755	1.093
CENPA	0.099	0.227	1.104	0.4361	0.6628	0.7076	1.7226
UPB1	-0.0584	0.0725	0.9433	-0.8061	0.4202	0.8184	1.0872
CABYR	0.1509	0.0752	1.1629	2.0063	0.0448	1.0035	1.3476
AFM	-0.0022	0.0609	0.9978	-0.0368	0.9707	0.8856	1.1242
HMMR	0.3139	0.1889	1.3687	1.6618	0.0965	0.9452	1.982
KIF20A	0.0587	0.2379	1.0604	0.2465	0.8053	0.6652	1.6904
PMAIP1	-0.1203	0.1857	0.8866	-0.648	0.517	0.6162	1.2758

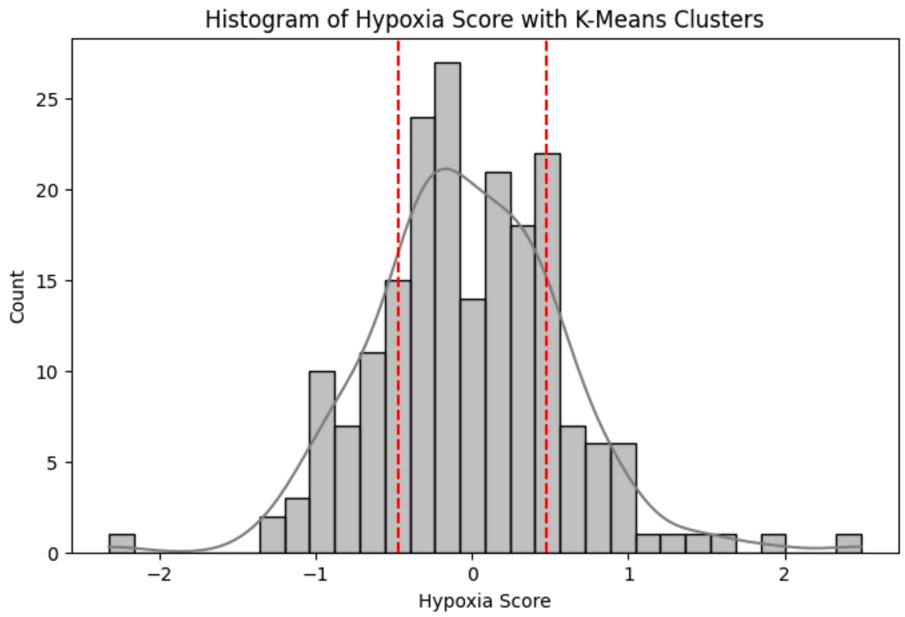

Figure S1. A histogram analysis confirmed a near-normal distribution of hypoxia risk scores.

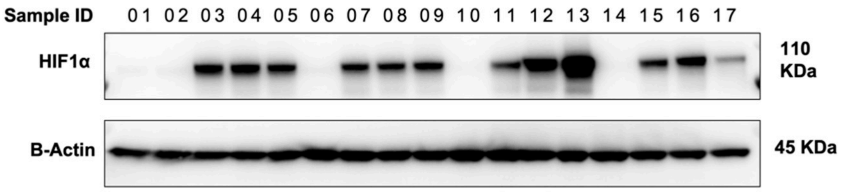

Cell	Sample ID	concentration of CoCl2 (μM)	Culture Timepoint
HepG2 (0.8M)	01	0	0h
	02	0	4 h
	03	100	
	04	200	
	05	400	
	06	0	8h
	07	100	
	08	200	
	09	400	
	10	0	24 h
	11	100	
	12	200	
	13	400	
	14	0	48h
	15	100	
	16	200	
	17	400	

Figure S2. The protein expression of HIF-1α on the Western Blot (WB) images under the conditions of different concentrations of $CoCl_2$ and different durations of hypoxia

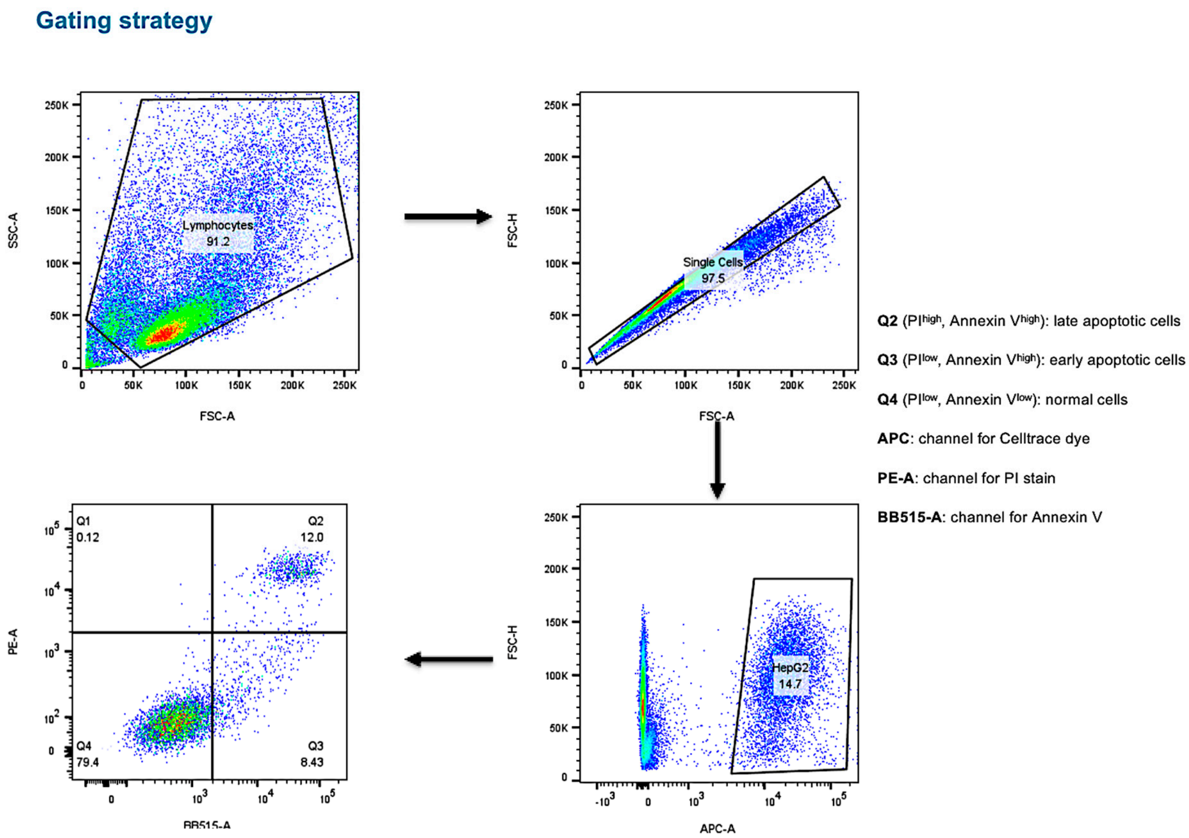

Figure S3. Gating strategy for apoptosis analysis by flow cytometry.
